# Outcomes of tunneled internal jugular venous catheters for chronic haemodialysis at the University College Hospital, Ibadan, Nigeria

**DOI:** 10.11604/pamj.2018.31.218.17525

**Published:** 2018-12-03

**Authors:** Yemi Raheem Raji, Samuel Oluwole Ajayi, Olusegun Aminu, Busayo Abiola, Oluwafemi Efuntoye, Babatunde Lawal Salako, Ayodeji Arije, Solomon Kadiri

**Affiliations:** 1 Department of Medicine, College of Medicine, University of Ibadan, Ibadan, Oyo state, Nigeria; 2 Department of Medicine, University College Hospital, Ibadan, Oyo state, Nigeria

**Keywords:** Catheter, complications, haemodialysis vascular access, Nigeria, outcomes

## Abstract

**Introduction:**

vascular access is an important aspect of haemodialysis treatments and determinant of patient outcomes. Arteriovenous (AV) fistula has been described as the preferred haemodialysis vascular access for patients on chronic dialysis. There continues to be a challenge with the creation of AV fistula, due to shortage of vascular surgeons skilled in the AV fistula creation particularly in source limited setting. We described the outcomes of the tunneled internal jugular venous catheters amongst our patients at the University College Hospital (UCH) Ibadan.

**Methods:**

a retrospective study of patients on maintenance haemodialysis at the UCH, Ibadan, we reviewed the records of all patients on chronic dialysis over a period of 5 years. Information obtained include demographics, types and aetiology of renal failure, types of vascular access, observed complications and outcomes.

**Results:**

a total number of 147 catheters were inserted during the period under review, 94 were males while 53 were females. The age range was 18-85 years while the mean age was 46.3 ± 17.2 years. The range and mean duration for Tunneled Dialysis Catheter (TDC) carriage were (30 - 1,440) and 220±185 days respectively. The observed immediate complications of TDCs were failed first attempt 7(4.7%), reactionary haemorrhage 5(3.4%), arrhythmia 3(2.0%), haemothorax 2(1.4%) while death during catheter placement was recorded in 2(1.4%) cases. Catheter related infection was the commonest long-term complications and occurred in 15 cases (10.1%), while being diabetic increased the risk of developing catheter related complications. One tenth of our patients with End Stage Renal Disease on TDC had kidney transplantation while catheter related mortality was 16.3%.

**Conclusion:**

internal jugular tunneled dialysis catheters despite its shortcomings, has been a safe procedure with good outcomes among our patients on maintenance haemodialysis.

## Introduction

Vascular access is an important aspect of haemodialysis treatment and arteriovenous (AV) fistula has been described as the most preferred haemodialyis access type for patients on chronic dialysis [1]. In addition, haemodialysis vascular access is a major determinant of outcomes among patients on maintenance haemodialysis [2, 3]. Over the last decade the National Kidney Foundation-Kidney Disease Outcome Quality initiatives (NKF-KDOQI) through the fistula first initiatives has spearheaded the campaign for initiating maintenance haemodialysis with AV fistula [4, 5]. The campaign is based on the several advantages of AV fistula over other forms of haemodialysis vascular access [6]. The benefits of AV fistula include low rate of bacteremia, thrombosis, stenosis and reduction in overall morbidity and mortality [3, 7]. Despite the success of the fistula first movement in ensuring that most patients with end stage renal disease (ESRD) commence haemodialysis with AV fistula, the use of Tunneled Dialysis Catheters (TDCs) is still on the rise worldwide [8, 9]. The use of dialysis catheters is often discouraged because of the various complications associated with its use, such complications include catheter related infections, thrombosis, subclavian or jugular vein stenosis, inadequate dialysis and poor quality of life [10, 11]. Furthermore, all-cause mortality is higher among End Stage Renal Disease (ESRD) patients dialyzing with dialysis catheter compared to patients undergoing dialysis with AV fistula [12]. Even with its many challenges, the use of TDCs is particularly common in the low and medium income countries (LMICs), where vascular surgeons skilled in AV fistula creation were not readily available and patients often present late [13]. Other group of patients on TDCs are End Stage Renal Disease (ESRD) patients with peripheral arterial disease, who are unsuitable for either of AV fistula or graft. Originally, TDCs were designed as short or medium term means of haemodialysis or long term vascular access pending the maturation of AV fistula or graft, or early kidney transplantation, but its use as a permanent vascular access in resource limited setting is now on the rise [13]. Nigeria with a population of over 180 million has a rising population of individuals with CKD and ESRD. However, most patients present very late to the hospital and usually commence haemodialysis with temporary haemodialysis vascular access [14, 15]. For those requiring maintenance haemodialysis, tunneled curved internal jugular catheter has progressively become the more commonly used vascular access [16, 17]. Despite this increased use of TDCs in the country, to our knowledge there has not been any comprehensive report on the safety, complications and economic cost of TDC. This study was designed to assess the utility and safety of tunneled curved internal jugular catheter use in patients with ESRD on maintenance haemodialysis.

## Methods

This is a retrospective study of ESRD patients on maintenance haemodialysis at the University College Hospital, Ibadan, Nigeria. We reviewed the dialysis records of all patients on chronic dialysis over a period of 5 years (January 2013-December 2017). Information obtained include demographics, types and aetiology of renal failure, types of vascular access, number of attempts at creating the vascular access, observed complications and outcomes. Maintenance haemodialysis was defined as regular intermittent haemodialysis offered to patients with ESRD [18]. All patients on maintenance dialysis with tunneled curved internal jugular catheter were observed for immediate and long term complications associated with the catheter use. The protocol used by our unit is as highlighted below.

### Procedure protocol

**Obtaining informed consent:** informed consent was obtained from all patients prior to the procedure and after explaining the procedure, why it is needed, possible complication that may arise from it and the post-catheter care required.

**Figure 1:**
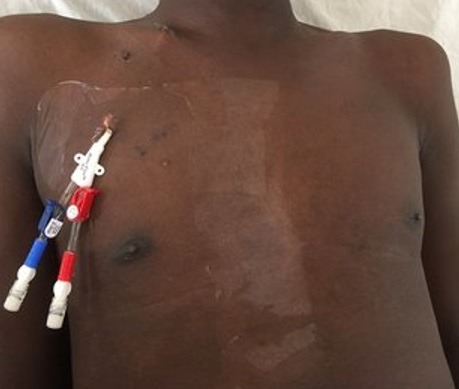
photograph of a patient with tunneled internal jugular haemodialysis catheter

**Dialysis catheter placement:** the tunneled internal jugular dialysis catheters were placed by the nephrologists or nephrology trainees under the supervision of the nephrologists. The right internal jugular vein was the preferred site of placement except where there were technical difficulties and was done under strictly an aseptic procedure. The approach for catheter insertion was modified Seldinger's technique, while ultrasonography guidance was only used in those in whom technical difficulty was encountered with the use of anatomical landmarks (Figure 1).

**Immediate complications of tunneled catheter insertions:** the patients were observed for 4-6 hours after the TDC placement to assess the occurrence of immediate complication, in addition to carrying out a post-catheter placement chest radiography. The latter was to ascertain the exact location of the catheter and exclude complications such as pneumothorax, haemothorax or lung contusion. Upon discharge, all patients were instructed to report any observed complications to the unit doctor on call, through a direct telephone line or call at the hospital.

**Care of haemodialysis catheter:** all patients had the care of the TDC before and after every dialysis with removal of blood clot and flushing of the catheter ports. The catheter arterial and venous ports were locked with heparinized saline (500 units/ml of unfractionated heparin) and 2.5mg/ml of gentamicin, each catheter port was locked with 1,000 units and 5mg of gentamycin after every session of haemodialysis. Those who developed catheter exit infections were treated with topical antibiotics while those with tunnel infection and bacteremia had courses of systemic antibiotics, in addition to removing the catheter.

**Late complications of tunneled internal jugular catheter use:** all patients were assessed once per week for complications relating to TDC or its use for haemodialysis during clinic visits and visits for haemodialysis.

**Statistical analysis:** data were analyzed using the Statistical Package for the Social Sciences (SPSS) for Windows version 20.0 International Business Machines Corporation, Armonk, New York (IBM, Armonk, NY). Estimates were expressed as either mean values with standard deviation for continuous variables, while categorical variables were expressed as proportions (percentage). Comparison for statistical significance was by independent Student's t-Test for continuous variables or chi-square for categorical variables. The level of significance was set at p ≤ 0.05.

## Results

**Figure 2:**
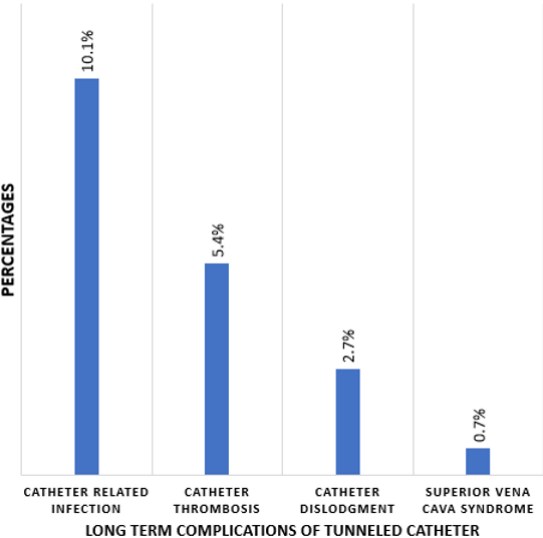
long term complications of tunneled internal jugular venous catheters

A total of 1170 patients had 5817 sessions of haemodialysis during the period under review, with an average of 5 sessions of haemodialysis per patient. Indications for dialysis were acute kidney injury (AKI) in 266 (22.7%), acute exacerbation of CKD in 496 (42.4%) and ESRD in 408 (34.9%) patients. Among patients with ESRD 147 (36.0%) had TDC placement and dialyzed with it, while 254 (62.3%) and 6 (1.4%) patients dialyzed with femoral catheters and arteriovenous fistula (AV) respectively. For individuals that had dialysis with TDC, 94 (63.9%) were males while 53 (36.1%) were females. The age was 18-85 years while the mean age was 46.3 ± 17.2years. The leading causes of ESRD were hypertension, chronic glomerulonephritis (CGN) and diabetes mellitus (DM) while the mean and range for the duration of TDC use were 220 ± 185 days and (30-1,440) days respectively (Table 1). Forty-eight (32.7%) patients had catheter related complications either during catheter insertion or while it was being used for dialysis. Immediate and late complications were observed in 20 (13.6%) and 29 (19.7%) patients respectively (Table 2). The immediate complications recorded were 7(4.7%) cases of failed first attempt, others were reactionary haemorrhage 5(3.4%), arrhythmia 3 (2.0%), haemothorax 2(1.4%), while death during catheter placement was recorded in 2 (1.4%) cases. Catheter site infection and catheter related bacteremia were the commonest long-term complications and it occurred in 15 cases (10.1%). Other chronic complications recorded were catheter thrombosis, dislodgement and superior vena cava syndrome (Figure 2). The commonest indications for catheter removal was death in 66 (44.7%) while kidney transplantation and occurrence of complications were the other reasons for discontinuation of catheter use (Table 2). Catheter related mortality was observed in 24 (16.3%) patients (Table 2). On univariate analysis, individuals with catheter related complications had a shorter mean duration of catheter carriage, while patients with diabetes mellitus were more likely to develop catheter related complications. Age and gender were not associated with duration of catheter carriage (Table 3).

**Table 1:** demographic characteristics of the end stage renal disease patients with tunneled internal jugular catheter for haemodialysis

Variables	Mean ± SD or percentage n = 147
Mean Age (years)	46.3 ± 17.2
Gender	
Female	53 (36.1%)
Male	94 (63.9%)
Etiology of ESRD	
Hypertension	44 (29.9%)
CGN	29 (19.7%)
DM nephropathy	22 (15.0%)
Obstructive uropathy	14 (9.5%)
HIVAN	8 (5.4%)
Others	15 (10.2%)
Unknown	15 (10.2%)
Mean BMI (kg/m^2^)	23.3 ± 4.4
Mean SBP (mmHg)	142.0 ± 28.3
Mean DBP (mmHg)	84.7 ± 15.6
Mean haemoglobin concentration (g/dl)	8.6 ± 3.2
Mean duration of catheter carriage (days)	220 ± 185
Mean time to events (complications) (days)	104.0 ± 113.2
Site of catheter	
Right internal jugular vein	146 (99.3%)

BMI – Body Mass Index, CGN - Chronic Glomerulonephritis, DBP – Diastolic Blood Pressure, DM – Diabetes mellitus, HIVAN – Human Immunodeficiency Virus Associated Nephropathy, SBP – Systolic Blood Pressure, SD – Standard Deviation

**Table 2:** complications and outcomes of tunneled internal jugular venous catheter placement and its use for hemodialysis

Complications/Outcomes	Percentage/proportion n = 147
Any complications	48 (32.7%)
Immediate complications	20 (13.6%)
Long term complications	29 (19.7%)
Catheter outcomes	
Patient alive and catheter functional	25 (17.0%)
Patient had kidney transplantation	19 (12.9%)
Removed due to complications	18 (12.2%)
Removed on request by patients	2 (1.4%)
Mortality	66 (44.8%)
Lost to follow up	17 (11.6%)
Patient outcomes	
Alive with catheter	21 (14.3%)
Alive with kidney transplantation	14 (9.5%)
Catheter related mortality	24 (16.3%)
Non – catheter related mortality	42 (28.6%)
Lost to follow up	32 (21.8%)

**Table 3:** demographic characteristics of the end stage renal disease patients with tunneled internal jugular catheter for hemodialysis

Variables	Patients with catheter related complications n = 48 Mean ±SD/frequency (%)	Patients without catheter related complication n = 99 Mean ±SD/frequency (%)	P-value
Mean Age (years)	47.5 ± 16.0	45.7 ± 17.8	0.56
**Gender**			
Female	21 (43.7%)	32 (32.3%)	0.18
Male	27 (56.3%)	67 (67.7%)	
**Etiology of ESRD**			
Hypertension	13 (27.1%)	31 (31.3%)	0.02
CGN	10 (20.8%)	19 (19.2%)	
DM nephropathy	12 (25.0%)	10 (10.1%)	
Obstructive	3 (6.25%)	11 (11.1%)	
Uropathy	1 (2.1%)	7 (7.1%)	
HIVAN	6 (12.5%)	9 (9.1%)	
Others	3 (6.25%)	12 (12.1%)	
**Unknown**			
Mean DBP (mmHg)	83.5 ± 14.7	84.8±15.9	0.73
Mean SBP (mmHg)	139.4 ± 30.6	143. ± 27.0	0.39
Mean BMI (kg/m^2^)	23.8 ± 4.3	23.0 ± 4.2	0.36
Mean haemoglobin concentration (g/dl)	8.5 ± 1.8	8.9 ± 2.3	0.45
Mean duration of catheter carriage (days)	186 ± 132	240 ± 210	0.01
Duration of catheter carriage > 180 days	21 (43.8%)	34 (34.3%)	0.27

BMI – Body Mass Index, CGN - Chronic Glomerulonephritis, DBP – Diastolic Blood Pressure, DM – Diabetes mellitus, HIVAN – Human Immunodeficiency Virus Associated Nephropathy, SBP – Systolic Blood Pressure, SD – Standard Deviation

## Discussion

Our study showed that TDC is increasingly being used as vascular access for maintenance haemodialysis in our hospital and one third of patients on TDC developed catheter related complications. The commonest immediate complication observed was failure of first attempt at passing the catheter, while catheter related infection was the leading long-term complications observed. There was a high rate of TDC use among our patients with ESRD, mainly for its convenience and ease of use and more importantly because its insertion can be carried out by the nephrologists, unlike the AV fistula creation that requires experienced vascular surgeons, whom were in short supply in most hospitals in Nigeria. Secondly, patients often present late in the hospital with advanced kidney disease [16, 19], such that most require immediate maintenance haemodialysis. This pattern of vascular access used for haemodialysis in our study is similar to what obtains in other LMICs, where vascular surgeons skilled in skills in AV fistula creation are not available [8, 20]. The increasingly important role of TDC for the delivery of haemodialysis in resource limited settings as against the use of AV fistula or graft is of great concern and could be a contributing factor to the poor quality of life and sub-optimal patients' outcomes. In one report on haemodialysis vascular access use from Uyo, South South Nigeria by Ekpe *et al.* [21], only 5% of their patients with ESRD dialyzed with permanent vascular access. Failed first attempt at catheter insertion was the commonest immediate complication observed among our patients, this is not surprising as the procedure was carried out without ultrasonographic guidance, due to lack of ultrasound scan machine in our dialysis centre at the time and non- availability of dedicated ultrasound machine for the procedure at the radiology department. The ultrasound assisted catheter insertion has been shown to reduce the rate of failed first attempts at catheter placement and other immediate complications commonly associated with blind insertion and has become standard of care [20, 22]. Two of our patients with TDC had haemothorax arising from catheter placement procedure and were detected through the post catheter insertion check radiograph. The two cases required reviews by the cardiothoracic surgeon, thoracotomy and under water sealed chest tube drainage. The use of ultrasonographic guidance and fluoroscopy may have reduced the risk of this complication [23-25]. Although some of our patients had reactionary haemorrhage around the catheter insertion and tunneling sites, none of them required blood transfusion solely because of this bleeding. The mortality of 2 in 147 catheter insertions observed in our study was higher compared to 1.25 in 1,000 catheter insertions reported by Vanholder *et al.* [26] among their patients, the use of ultrasound guidance may be responsible for the lower mortality in their series. The 2 mortalities recorded during catheter insertions in our patients were suspected to be due to arrhythmias during guidewire or catheter introduction. The occurrence of arrhythmias during jugular venous catheter insertion buttresses the need to provide continuous cardiac monitoring during and immediately after catheter insertion. A thorough cardiac evaluation prior to the catheter insertion will also guide patient's selection for the procedure and reduce the incidence of arrhythmias.

The leading long-term complication among our patients was catheter related infection (10.1%) and this was high compared to 0.55% reported by Katneni *et al.* [27]. The high rate of infection among our patients could be explained by the differences in frequency of catheter care, since most of our patients rarely dialyzed more than once a week, whereas ESRD patients in the Katneni *et al.* [27] series were dialyzing thrice a week, during which catheter care were carried out as well. Half of the catheter related infection among our patients were bacteremia and warranted removal of the catheter. The observed high rate of catheter related infection was despite locking the catheter ports with gentamycin after each session of haemodialysis. Locking the arterial and venous catheter ports with antibiotics has been shown to reduce the incidence of catheter related infection, particularly bacteremia [28], suggesting that the incidence of catheter related infections would have been higher in our patients, if not for the use of antibiotic catheter lock or perhaps the antibiotic use was inappropriate [29]. The high rate of catheter related infection in our patients was similar to those reported in other forms of in-dwelling catheter use in our setting. Ademola *et al.* [30] reported peritonitis in 10 out 27 children who had peritoneal dialysis using improvised catheter in Ibadan while Komolafe *et al.* [31] observed catheter related infection rate of 19.4% in children who had ventriculoperitoneal shunts for treatments of hydrocephalus in Ile-Ife. Only 10% of our ESRD patients who were on TDC for haemodialysis had their catheter removed because they had kidney transplantation. This is because only few of our patients could afford kidney transplantation as a modality of treatment, as patients pay out of pockets for their renal care in Nigeria [32]. The inclusion of renal care services in the current National Health Insurance Scheme (NHIS) will go a long way in ensuring more patients transit from TDC based haemodialysis to kidney transplantation. We observed that ESRD patients with diabetes mellitus were more likely to develop catheter related complications and this agrees with previous reports on TDC use. Uncontrolled diabetes mellitus increases the risk of infection, thrombosis and catheter failure [33, 34]. This is because hyperglycaemia provides a good medium for bacteria growth, in addition to immunosuppression and vascular disease commonly encountered in patients with uncontrolled diabetes mellitus. The catheter related mortality of 16.3% observed in this cohort was high, however, the high mortality might have also been contributed to by other factors. Other factors contributing to the poor patient outcomes were inadequate haemodialysis, sub-optimal anaemia treatment, cardiovascular mortality, malnutrition and high incidence of infection. Despite the low rate of complications in the use of TDC, its use should be restricted to when the ideal is not available, particularly in the setting where the technical know-how for AV fistula creation is not readily available. However, the use of TDC in resource challenge setting should be embarked upon with adequate precautions geared towards reducing the high rate of complications. These steps should include appropriately selecting suitable patients for the procedure, use of ultrasonography and fluoroscopy, cardiac monitoring during and immediately after the procedure, while adequate catheter care during and in-between dialysis must be ensured. While TDC is being used as a temporary vascular access, efforts should be made to train vascular surgeons in creation of AV fistula in source challenged country like Nigeria. This study is not without limitations, some patients with TDC were lost to follow up, such that the patients' and catheter outcomes could not be ascertained in them. Also, the contributions of other factors such as inadequate anaemia treatment and haemodialysis could not be excluded in the patients with catheter related mortalities.

## Conclusion

Internal jugular tunneled dialysis catheters despite its shortcomings, has been a safe procedure with good outcomes among patients on maintenance haemodialysis. Despite its usefulness tunneled dialysis catheter should still serve as a stop gap vascular access pending the creation of AV fistula or early kidney transplantation. The use of ultrasonographic guidance, fluoroscopy and cardiac monitoring during catheter insertion, in addition to prior thorough cardiovascular evaluation are measures that will improve the catheter and patient outcomes.

### What is known about this topic

- Most patients with chronic kidney disease in sub-Saharan African countries present late to the hospital, often at a time when dialysis is required;
- Haemodialysis as a modality of treatment of end stage renal disease is becoming increasingly available in sub-Saharan Africa;
- Most patients in sub-Saharan African countries dialyzed with catheters rather than the arteriovenous fistula which is the preferred vascular access that improves patient's survival, because vascular surgeons skilled in arteriovenous fistula creation are not readily available.

### What this study adds

- The use of tunneled haemodialysis catheter is associated with relatively high rate of complications;
- Tunneled dialysis catheter should only be used where access to arteriovenous fistula creation is not readily available and necessary precautions should be taken to reduce the risk of complications;
- There is need for training of vascular surgeons in arteriovenous fistula creation in resource challenged setting in order to reduce significantly the use of tunneled dialysis catheter and its associated complications.

## Competing interests

The authors declare no competing interests.
